# Syndrome de la queue de cheval révélant une hydatidose vertébro-médullaire

**DOI:** 10.11604/pamj.2020.36.225.21606

**Published:** 2020-07-28

**Authors:** Imane Benabdallah Staouni, Zineb Marzouki, Meryem Haloua, Youssef Alaoui Lamrani, Meryem Boubbou, Mustapha Maâroufi, Badr-eddine Alami

**Affiliations:** 1 Service de Radiologie, Centre Hospitalo-universitaire Hassan II, Fès, Maroc

**Keywords:** Hydatidose, rachis, IRM, chirurgie, pronostic, Hydatid disease, spine, MRI, surgery, prognosis

## Abstract

L´hydatidose est une anthropozoonose due au développement chez l´homme de la forme larvaire de l´Echinococcus granulosus. Elle sévit surtout dans les régions rurales et d´élevages au niveau du bassin méditerranéen, l´Amérique du sud, proche et moyen orient. La localisation vertébrale est rare mais représente la forme la plus grave des localisations osseuses de l´hydatidose. Elle touche préférentiellement le rachis dorsal. Cette atteinte est grave vu le risque de compression médullaire. Nous illustrons le cas d´une patiente de 60 ans, admise pour lombo-radiculalgies paralysantes bilatérales mal systématisées, d´évolution progressive, associées à une impériosité mictionnelle. L´IRM a retrouvé une hydatidose vertébrale lombaire infiltrant les structures endo et extra-canalaires et comprimant les racines de la queue de cheval. La patiente a été opérée par voie postérieure et l´évolution a été favorable.

## Abstract (en)

Hydatid disease is an anthropozoonosis caused by the larval stages of the genus Echinoccus granulosus in humans. It mainly occurs in subjects living in the rural and livestock areas in the Mediterranean basin, South America, Near and Middle East. Spinal hydatid disease is rare but it is the most severe form of bone hydatid disease. It mostly affects the dorsal spine, increasing the risk of spinal injury; hence its severity. We here report the case of a 60-year-old female patient admitted with progressive bilateral, poorly systematized, paralyzing lumbar radiculopathy associated with urinary urgency. MRI showed lumbar spinal hydatid disease infiltrating the intra and extra-ductal structures and compressing the roots of the horse tail. The patient underwent surgery through a posterior approach with favorable outcome.

## Introduction

L´hydatidose osseuse est une affection rare (0,5 à 2%). Les localisations rachidiennes sont les plus fréquentes (44%) et les plus graves [1]. L´évolution clinique est souvent latente, ce qui pose le problème de retard diagnostique et de difficulté thérapeutique.

## Patient et observation

**Figure 1:**
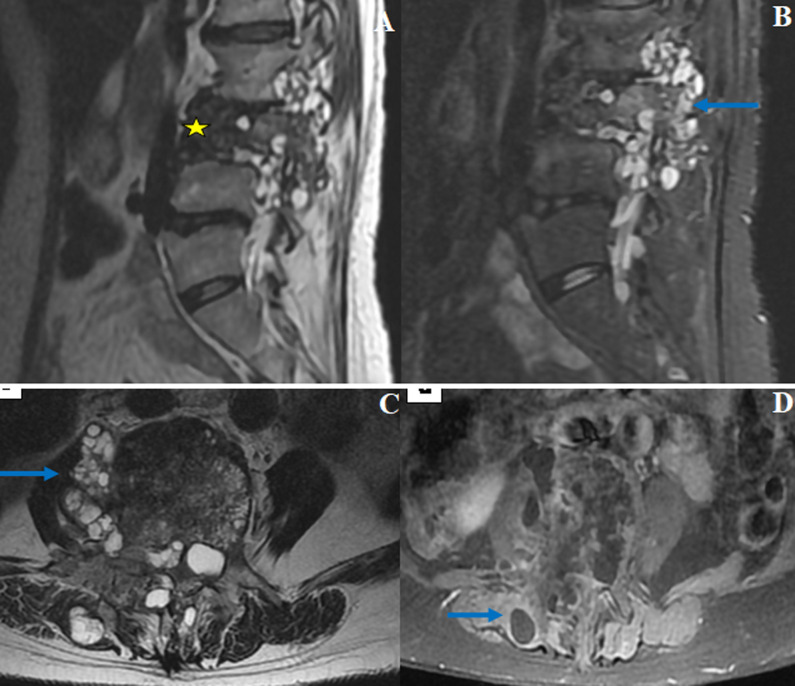
IRM en coupe sagittale T2 (A), sagittale STIR (B), axiale T2 (C) et axial T1 (D) avec gadolinium montrant un tassement en galette du corps vertébral de L3 (étoile), avec de multiples lésions vésiculaires kystiques en hypersignal T2 et STIR, non rehaussées après contraste, de siège intracanalaire, au niveau des trous de conjugaison et au niveau des parties molles péri et para-vertébrales (flèches)

Il s´agit d´une patiente âgée de 60 ans, sans antécédents pathologiques notables, admise aux urgences neurochirurgicales pour des lombo-radiculalgies paralysantes bilatérales mal systématisées, d´évolution progressive, associées à une impériosité mictionnelle. L´examen clinique a trouvé une patiente consciente, présentant une paraparésie grade D de Frankel prédominant au niveau distal, avec abolition bilatérale des réflexes ostéo-tendineux rotuliens et achilléens. L´examen de la sensibilité a retrouvé une hypoesthésie périnéale. Le testing musculaire a trouvé une force musculaire cotée à 3/5 aux niveaux du muscle triceps sural, jambier antérieur et fléchisseurs plantaires des deux côtés. Devant ce tableau clinique évoquant un syndrome de la queue de cheval, une IRM lombaire a été réalisée en séquences pondérées T1, T2, STIR et T1 avec Gadolinium dans les plans axial et sagittal. Elle a mis en évidence la présence de tassement en galette des corps vertébraux de L3 et L4, apparaissant en hétérosignal T1 et T2, alors que le disque intervertébral présentait un signal normal. Il s´y associe plusieurs formations kystiques, de taille variable, intravertébrales décrites en hyposignal T1, hypersignal T2, non rehaussées après injection du contraste. Ces lésions présentaient une extension intracanalaire, responsables ainsi d´une compression des racines de la queue de cheval. Il s´y associe d´autres lésions kystiques des parties molles péri-vertébrales, du muscle psoas droit et des muscles paravertébraux (Figure 1). L´ensemble de ces anomalies radiologiques faisait évoquer le diagnostic d´hydatidose vertébro-médullaire.

Un bilan d´extension, fait de radiographie thoracique et d´échographie abdominale, à la recherche d´autres localisations hydatiques a été réalisé et revenu négatif. Le bilan biologique était sans particularités. La patiente a été opérée par voie postérieure avec réalisation d´une laminectomie de L3 et L4, évacuation des différents kystes et décompression de la queue de cheval associée à une ostéosynthèse L2-L5 par un matériel type Cotrel Dubousset. En peropératoire, les muscles paravertébraux et les berges de la laminectomie ont été couverts de compresses imbibées de solution scolicide, type sérum salé hypertonique. Le diagnostic d´hydatidose vertébro-médullaire a été retenu et confirmé à l´étude anatomopathologique après exérèse chirurgicale. Les suites opératoires immédiates ont été marquées par un début de récupération neurologique et la patiente a été mise sous traitement médical antiparasitaire à base d´albendazole 400 mg deux fois par jour.

## Discussion

L´hydatidose osseuse est rare même dans les pays d´endémie. La localisation rachidienne étant la plus fréquente. Le rachis dorsal est le plus touché, représentant 80% des localisations rachidiennes alors que la localisation lombo-sacrée constitue 18 % des cas [2]. Elle survient essentiellement chez le sujet jeune [3]. Sur le plan clinique, l´état général reste longtemps conservé, la symptomatologie neurologique est souvent faite de rachialgies, troubles moteurs et/ou sensitifs et troubles sphinctériens en rapport avec la compression médullaire. Notre patiente présentait des lombo-radiculalgies paralysantes bilatérales, d´aggravation progressive avec à l´examen clinique une hypoesthésie en selle et abolition des réflexes ostéo-tendineux aux deux membres inférieurs. Les collections paravertébrales peuvent aussi constituer un mode de révélation de l´hydatidose rachidienne, sous forme de tuméfaction locale fistulisée ou non. L´éosinophilie sanguine est dans la plupart des séries peu élevée et inconstante [4]. Notre patiente ne présentait pas une éosinophilie biologique. L´atteinte rachidienne est souvent primitive, alors que les lésions radiculo-médullaires sont secondaires à la migration des vésicules hydatiques à travers les trous de conjugaison ou à des destructions osseuses et compression des structures neurologiques [5]. C´est le cas de notre patiente qui présentait une extension intracanalaire des formations vésiculaires via les trous de conjugaison.

L´IRM constitue l´examen de choix pour l´exploration des lésions rachidiennes et médullaires, notamment devant un tableau de compression médullaire. Elle permet de préciser le siège exact de l´atteinte (osseux, épidural, intradural, intramédullaire) et l´extension aux parties molles péri-vertébrales. Par ailleurs, elle permet de repérer les signes de souffrance médullaire, d´orienter le geste chirurgical et joue également un rôle important dans la surveillance postopératoire. Les vésicules hydatiques apparaissent en hyposignal T1, hypersignal T2, généralement non modifiés après injection du contraste. Par contre, un rehaussement des parois et des cloisons peut se voir si les kystes sont remaniés [6]. Dans l´hydatidose rachidienne, le disque est longtemps conservé contrairement à la spondylodiscite infectieuse qui constitue un diagnostic différentiel. Chez notre patiente, l´analyse du disque vertébral a objectivé un signal normal ce qui a permis d´écarter le diagnostic de spondylodiscite. Le traitement de l´hydatidose rachidienne repose sur la chirurgie qui doit être aussi radicale que possible. Néanmoins, cet objectif demeure illusoire vu l´extension souvent diffuse des lésions au moment du diagnostic et le risque important de récidives. Le traitement médical antiparasitaire (à base d´Albendazole ou de Mebendazole) est réservé aux formes inopérables ou de mauvais pronostic, mais aussi indiqué comme thérapeutique adjuvante de la chirurgie pour minimiser le risque de récidive [7].

## Conclusion

L´hydatidose vertébro-médullaire est une entité rare même dans les pays d´endémie. Elle est très agressive avec un haut risque de compression médullaire. L´IRM est l´examen de choix réalisé en urgence devant tout tableau clinique de compression radiculo-médullaire. Le traitement est essentiellement chirurgical. Le risque de récidive étant important d´où la nécessité des mesures de prévention et d´éducation sanitaire dans les pays endémiques.
